# Protein markers of dysfunctional HDL in scavenger receptor class B type I deficient mice

**DOI:** 10.1186/s12967-018-1502-y

**Published:** 2018-06-07

**Authors:** Jia Cao, Yanyong Xu, Feifei Li, Liang Shang, Daping Fan, Hong Yu

**Affiliations:** 1 Department of Biochemistry and Molecular Biology, Hubei Provincial Key Laboratory of Developmentally Originated Disease, Wuhan University School of Basic Medical Sciences, 185 Donghu Road, Bldg. 2, 2-206, Wuhan, 430071 China; 2 Department of Cell Biology and Anatomy, School of Medicine, University of South Carolina, Columbia, SC USA

**Keywords:** High density lipoprotein, Dysfunction, Protein markers, SR-BI, Probucol

## Abstract

**Background:**

Scavenger receptor class B type I (SR-BI) plays a key role in high density lipoproteins (HDL) metabolism. SR-BI deficiency in mice results in enhanced susceptibility to atherosclerosis with abnormal large, cholesterol enriched, and functional impaired HDL. This study was to characterize the protein markers of dysfunctional HDL in SR-BI deficient (SR-BI^−/−^) mice and to test if the defective of HDL might be affected by probucol treatment.

**Methods:**

Shotgun proteomics and 2-D gel electrophoresis were performed to examine the profile of HDL protein and distribution of HDL particles isolated from SR-BI^−/−^ mice. HDL’s cell-function, paraoxonase 1 (PON1) and myeloperoxidase activity were assessed. The mice were treated with 1.2 mg/g/day probucol for 6 weeks and the impact on HDL protein markers was analyzed. The differential proteins were quantified by Western blotting.

**Results:**

The relative amount of protein in SR-BI^−/−^ HDL was decreased by about 25% compared to that in HDL from wild type (WT) mice. Compared to WT HDL, relative protein abundance of representative apoAI and PON1 in SR-BI^−/−^ HDL were significantly reduced, whereas acute-phase protein serum amyloid A (SAA) and apoAIV, proteinase inhibitor proteins α-1-antitrypsin (A1AT) were increased. The distribution of plasma apoAI-containing HDL particles in SR-BI^−/−^ mice was also dramatically altered, although plasma apoAI level was no difference. The protein alterations were accompanied with dysfunction of SR-BI^−/−^ HDL, evidenced by impaired cholesterol homeostasis in macrophages, and reduced anti-oxidative and anti-inflammatory effects. Probucol treatment of SR-BI^−/−^ mice could restored the relative contents of critical proteins including apoAI, PON1, SAA, apoAIV and A1AT on HDL, and improve HDL dysfunction despite decreased HDL-C level.

**Conclusion:**

SR-BI deficiency leading to dysfunctional HDL is closely related to alteration of HDL protein, suggesting that identification of apoAI, PON1, SAA, apoAIV, and A1AT may serve as the valuable protein markers for diagnosis and therapeutics of dysfunctional HDL-related metabolic diseases.

**Electronic supplementary material:**

The online version of this article (10.1186/s12967-018-1502-y) contains supplementary material, which is available to authorized users.

## Background

Plasma high-density lipoprotein (HDL) is a well-established cardiovascular protective factor with multiple functions, including mediating reverse cholesterol transport, and exerting anti-oxidative, anti-inflammatory, anti-thrombotic and vasodilatory activities [1]. Epidemiological and clinical studies have shown that HDL cholesterol (HDL-C) level is inversely correlated with coronary heart disease risk [2]. However, nearly half of cardiovascular clinical events occurred in subjects with normal or even high levels of HDL-C [3]; and clinical trials with drugs that raised HDL-C levels by cholesteryl ester transfer protein (CETP) inhibitors, had failed to reduce the risk of cardiovascular events [4]. These controversial findings suggest that HDL-associated functional factors, instead the levels of HDL-C, are important for the antiatherogenic properties. Indeed, it has been known that HDL particles have substantial compositional heterogeneity in its lipids, apolipoproteins, lipid transfer proteins, enzymes and numerous other proteins under pathophysiological conditions because of their complex metabolic function. The evidence from transgenic mice indicates that protein composition determines the antiatherogenic properties of HDL. Alterations in protein composition involved in HDL metabolism could promote atherosclerosis, even if plasma HDL-C was normal or elevated [5]. Recent studies come up with a new concept of “dysfunctional HDL”, which suggests that antiatherogenic HDL can be converted to proatherogenic HDL under dyslipidemic and inflammatory states in metabolic diseases, and thus may accelerate atherosclerosis [6]. Therefore, the assessment of dysfunctional HDL by measuring the protein composition, and the identification of biomarkers of dysfunctional HDL in different experimental models, will provide important information for the evaluation of cardiovascular risk in patients and for the development of new antiatherogenic therapies [7].

Scavenger receptor class B type I (SR-BI), a well-defined HDL receptor, is a prominent regulator of HDL metabolism, which controls the plasma HDL-C level. It mediates the selective uptake of cholesteryl esters from HDL by the liver and steroidogenic organs, and facilitates the efflux of cholesterol from peripheral tissues to HDL [8]. It has been reported that several mutations in human SR-BI gene, which are associated with low SR-BI protein levels, caused elevated plasma HDL-C levels, a reduction in cholesterol efflux from macrophages, altered platelet function, and decreased adrenal steroidogenesis [9–11]. The SR-BI deficient (SR-BI^−/−^) mice showed a nearly twofold increase in plasma total cholesterol (TC) and HDL-C levels, accompanied with impaired anti-oxidative function, and lipid deposition in the aorta, contributing to the enhanced susceptibility to atherosclerosis [12–14]. However, the protein composition of defective HDL in SR-BI^−/−^ mice has not been well studied.

Therefore, in this study we used shotgun proteomics, a powerful tandem mass spectrometry (MS/MS) approach following the protein separation by liquid chromatography (LC) [15], to compare the protein composition of HDL particles isolated from SR-BI^−/−^ mice and wild type (WT) mice. Meanwhile, HDL-mediating cholesterol flux in macrophages, anti-oxidative and anti-inflammatory function of HDL were detected to verify the functional status of HDL. Considering that probucol as a cholesterol-lowering and antioxidant drug, could alter the abnormal HDL-C level in SR-BI^−/−^ mice [16], probucol treatment was used to investigate whether the alteration of protein markers and HDL function could be affected in SR-BI^−/−^ mice. The goal of this study is to quantify the protein characteristics of SR-BI^−/−^ HDL, providing the valuable protein markers for diagnosis and therapeutics of dysfunctional HDL-related metabolic diseases.

## Methods

### Mice

C57BL/6 WT mice and apolipoprotein E deficient (apoE^−/−^) mice were obtained from Vital River Laboratory Animal Technology Company, China. SR-BI heterozygous (SR-BI^+/−^, 1:1 mixed C57BL/6 × S129 genetic background) mice were kindly provided by Professor MacRae F. Linton (Vanderbilt University, USA). SR-BI^−/−^ mice were generated from heterozygous mating pairs or by mating female SR-BI^+/−^ with SR-BI^−/−^ males. SR-BI genotypes were determined by PCR analysis of DNA extracted from tail biopsies [17]. All of the mice were housed in microisolator cages on a rodent chow diet. Animal care and experimental procedures were performed under the regulations of the Institutional Animal Care and the Ethics Committee for Animal Experiments of Wuhan University.

### Isolation of plasma HDL and determination of HDL composition

Blood samples from WT (SR-BI^+/+^) or SR-BI^−/−^ mice (both male and female) were collected with heparin collection tube by retro-orbital venous plexus puncture after overnight fasting. The plasma was immediately separated by centrifugation at 4000×*g* for 10 min at 4 °C. HDLs were isolated from pooled plasma samples by ultracentrifugation described in our previous work [18]. Briefly, apoB containing lipoproteins in plasma were precipitated by the addition of 20% polyethylene glycol (PEG) in 200 mM glycine (pH 10.0, plasma/PEG ratio, 1.0: 0.04), then the HDL-containing supernatant was dialyzed against 0.9% NaCl, 0.3 mM EDTA (pH 8.0), and the HDL was further isolated at density < 1.21 g/mL by ultracentrifugation, desalted and concentrated in phosphate-buffered saline (PBS) to a final concentration of 1 μg/µL.

Total cholesterol (TC), free cholesterol (FC) and triglyceride (TG) levels in HDL were determined enzymatically using Cholesterol and Triglycerides GPO Reagent kits (Raichem, USA). Protein concentration of HDL was determined by Lowry DC kit (BioRad, USA). HDL phospholipid was measured using Wako Phospholipids B enzymatic kit (Wako Laboratory Chemicals, USA). HDL composition was analyzed by calculating the percentage of TC, FC, TG, phospholipid, and total proteins.

### Shotgun LC–MS/MS

LC–MS/MS (liquid chromatography–tandem mass spectrometry) was performed at State Key Laboratory of Proteomics in the Beijing Proteome Research Center. Equal protein amounts (50 µg) of HDL preparations were separated by 10% SDS-PAGE (120 V, 20 min), and the totality of each lane was then sliced into 10 bands followed by in-gel digestion with trypsin. The resulting peptides were analyzed by reverse phase liquid chromatography coupled with tandem mass spectrometry (RP LC–MS/MS) on an LTQ-Orbitrap Velos mass spectrometer (Thermo Electron, San Jose, CA). All MS/MS spectra were identified by using Sorcerer-SEQUEST Algorithm version 4.0.3 (Sage-N Research, San Jose, CA) against the Refseq mouse protein sequence database. The searching parameters were set up as follows: partial trypsin (KR) cleavage with two missed cleavage was considered; the variable modification was oxidation of methionine; carbamidomethylation was set as fixed modification; the peptide mass tolerance was 20 ppm, and the fragment ion tolerance was 1 Da. Functional annotation of proteins detected in HDL was obtained from UniProt (http://www.uniprot.org/). Spectral counts were recorded to quantify relative protein abundance. This approach is based on the correlation between protein abundance and spectral counts. The higher abundance a particular protein is, the more MS/MS spectra are collected for peptides of that protein [19].

### Western blotting analysis

To identify the levels of specific proteins in HDL or plasma of SR-BI^−/−^ mice, we further used Western blotting to quantify the different proteins detected in the proteomic analysis. Appropriate amount of HDL proteins (2 µg HDL proteins for checking apoAI, and 15 µg HDL proteins for checking other proteins) or mouse plasma (0.5 µL plasma for checking apoAI and paraoxonase 1 (PON1), 1 µL plasma for checking other proteins) was loaded and separated by 10% SDS-PAGE, and transferred onto nitrocellulose membranes, then target proteins were detected by primary antibodies including goat anti-apoAI (Ab7614), mouse anti-PON1 (Ab24261), α-1-antitrypsin (A1AT) rabbit (Ab133642) antibodies from Abcam, goat anti-apoAIV (Santa, SC-19036), and rat anti-SAA antibodies (Santa, SC-59680), and HRP-conjugated secondary antibody. Signal was detected using an enhanced chemiluminescence (ECL) kit.

## 2D gel electrophoresis

ApoAI-containing HDL distributions of SR-BI^+/+^ and SR-BI^−/−^ mice were analyzed by 2D nondenaturing agarose-polyacrylamide gel electrophoresis (2DE) modified from Asztalos et al. [18, 20]. Briefly, in the first dimension, 2 µL of fresh plasma lipoproteins were separated on 0.75% agarose gel. Individual agarose strips were transferred to the top of 3–16% nondenaturing polyacrylamide gradient gels and separated in the second dimension. Lipoproteins were transferred to 0.2 µm nitrocellulose membranes, and 2DE patterns of apoAI-containing subpopulations were detected by rabbit anti-mouse apoAI primary and HRP-conjugated secondary antibody. Signal was detected using an ECL kit.

### Measurement cholesterol content in macrophage

Mouse peritoneal macrophages from WT C57BL6 mice were harvested by peritoneal lavage 3 days after intraperitoneal injection of 2.5 mL of 3% thioglycollate. Macrophages were suspended in 5% FBS/DMEM and seeded onto 6-well plates at a density of 1 × 10^6^ cells/well. After 3 h, non-adherent cells were removed by washing with DMEM, the macrophages were incubated for 48 h in DMEM alone or containing either SR-BI^+/+^ HDL or SR-BI^−/−^ HDL (12.5, 25, 50 µg protein/mL), then washed twice with DPBS and air dried. Cell lipids were extracted by overnight incubation at room temperature in isopropanol. The FC and TC contents in the lipid extracts were then measured by Cholesterol Reagent (Raichem). Cell proteins were solubilized by adding 1 N NaOH to the wells, and the protein concentration was measured by Lowry DC protein kit (BioRad, USA).

### PON1 activity and MPO activity assays

The measurement of PON1 activity in plasma or HDL particle using paraoxon (Sigma, USA) as substrate was described in our previous work [21]. One unit of PON1 activity in serum or HDL was defined as 1 nmol of p-nitrophenol formed per minute under the definition conditions and expressed as U in per ml serum or per mg HDL. Myeloperoxidase (MPO) activity was measured by a MPO determination kit (Jiancheng Biotech Ltd, China), according to the manufacturer’s instructions.

### Determination of HDL oxidative/anti-oxidative properties by cell-free assay

To determine the anti-oxidative properties of HDL, the cell-free assay was used with modifications [22, 23], using apoE^−/−^ mice plasma as the fluorescence-inducing agent. HDL was isolated by ultracentrifugation or from plasma after apoB-lipoprotein precipitation using dextran sulfate [23]. The change in fluorescence intensity as a result of the oxidation of dichlorodihydrofluorescein (DCFH) by apoE^−/−^ mice plasma in the absence or presence of HDL was tested. Briefly, 0.3–0.5 µL plasma from apoE^−/−^ mice and 15 µL of isolated HDL (final concentration of 15 µg/mL) or 15 µL of HDL-containing dextran sulfate supernatant were incubated with PBS to a total volume of 200 µL per well in black polystyrene microplates. After 90 min of incubation on a rotator at 37 °C, 2 µL of 2.0 mg/mL fresh DCFH-DA in methanol solution was added to each well, and incubated for additional 30 min with rotation. Fluorescence signal was determined (excitation/emission wavelength = 485 nm/530 nm, cutoff of 515 nm) using a Fluorescent Plate Reader (GENIOS VA2000 plate reader, TECAN). Assay was performed in triplicates and values in the absence of HDL were normalized to 1. After the addition of the test HDL, values < 1.0 indicated anti-oxidative HDL, values > 1.0 indicated oxidative HDL.

### Anti-inflammatory activity of HDL in oxLDL-induced macrophages

Human LDLs (Prospec-Tany company, Israel) was incubated with 10 µmol/L CuSO_4_ for 24 h at 37 °C, then dialysis with PBS and obtained the oxidized LDL (ox-LDL). Peritoneal macrophages from WT mice were cultured in 6-well plates, treated with or without 25 µg/mL ox-LDL, containing SR-BI^+/+^ HDL or SR-BI^−/−^ HDL (50 µg/mL) in DMEM, for 6 h at 37 °C. Afterwards, the supernatants were collected by centrifuged at 12,000 rpm to remove debris, then stored at − 80 °C until cytokine analysis. Monocyte chemotactic protein 1 (MCP-1) and tumor necrosis factor α (TNF-α) were quantified using ELISA kits (eBioscience) according to the manufacturer’s instructions.

Meanwhile, the cultured macrophages were collected using Trizol reagent, total RNA of macrophages was isolated and reverse transcribed into cDNA with reverse transcriptase (Invitrogen, USA). The target region in mouse MCP-1 and TNF-α gene was amplified by quantitative real-time PCR (Q-PCR) using universal PCR master mix (Invitrogen, USA). The primers for MCP-1 were 5′-AGGTCCCTGTCATGCTTCTG-3′ and 5′-TCTGGACCCATTCCTTCTTG -3′. The primers for TNF-α were 5′-CGTCAGCCGATTT GCTATCT -3′ and 5′-CGGACTCCGCAAAGTCTAAG -3′. The primers for 18s rRNA were 5′-CGCGGTTCTATTTTGTTGGT-3′ and 5′-AGTCGGCATCGTTTATGGTC-3′. The relative expression levels of these genes were detected by normalization of the cycle threshold (Ct) to that of the control 18S RNA. 2^−∆∆Ct^ was used to represent the relative expression of target genes.

### Effects of probucol treatment on SR-BI^−/−^ mice

Eight male SR-BI^−/−^ mice at 19–20 weeks of age (body weight: 22.7 ± 1.1 g) were randomly divided into control group and probucol treatment group. All mice were fed a normal chow diet. Probucol (1.2 mg/g/day) was intragastrically given to SR-BI^−/−^ mice for 6 weeks and 0.5% CMC-Na as the control. The levels of plasma TC and TG were determined enzymatically. Lipoprotein cholesterol profiles were made by fast protein liquid chromatography (FPLC) as described our previous study [18]. The expression levels of specific plasma HDL-associated proteins in SR-BI^−/−^ mice after probucol treatment were detected by Western blotting. Plasma PON1 activity, MPO activity and oxidative/anti-oxidative properties of HDL were measured using above methods.

### Statistical analysis

Statistical analysis was performed using Prism 5. Continuous variables were expressed as mean ± SD (standard deviation). The two-tailed Student’s *t* test for parametric variables was used to assess difference between two groups. *P *< 0.05 were considered statistically significant.

## Results

### Lipid and protein contents in SR-BI^−/−^ HDL

HDLs isolated from WT (SR-BI^+/+^) and SR-BI^−/−^ mice by ultracentrifugation were analyzed for the composition of lipids and proteins. In lipid classes, SR-BI^−/−^ HDL displayed a significantly increased percentage of FC and TC. FC content increased to 20.90% by 3.7-fold as compared with only 4.42% FC in SR-BI^+/+^ HDL, and TC content increased from 18.83% (SR-BI^+/+^ HDL) to 29.11% (SR-BI^−/−^ HDL); while phospholipid content was not significantly altered. The relative amount of protein in SR-BI^−/−^ HDL was decreased by about 25% compared with SR-BI^+/+^ HDL (Additional file 1: Table S1), suggesting that SR-BI deficiency in mice markedly affected HDL protein and lipids ratio.

### Proteomic alterations of SR-BI^−/−^ HDL

To identify HDL-associated proteins, we performed proteomic analysis of purified HDL using LC–MS/MS. After removing predicted contaminants such as keratin, 50 HDL-associated proteins in WT mice and 78 proteins in SR-BI^−/−^ mice were identified as true-positives (proteins were required to be present at > 0.05% of the total spectral counts). To assess the alterations of the contents of the proteins involved in HDL function, identified HDL-associated proteins were grouped into functional categories. The result showed that some proteins involved in immune response like beta-2-microglobulin, some in cell adhesion such as insulin-like growth factor-binding protein, integrin αIIb, integrin β1, and some others in proteinase inhibition such as antithrombin-III, were only been identified in SR-BI^−/−^ HDL, but not in SR-BI^+/+^ HDL. As the majority of HDL-associated proteins were common to both control SR-BI^+/+^ and SR-BI^−/−^ HDL, relative protein abundance was quantified by spectral counts, and resulting outputs were expressed as a percentage of the total spectral counts obtained per group. The analysis revealed that among the 78 proteins identified in SR-BI^−/−^ HDL, 26 proteins were appeared or increased and 10 proteins were decreased, when compared to those in SR-BI^+/+^ HDL. The relative levels of apoAI, apoAII, apoCI, apoCII, apoM and PON1 were significantly reduced, whereas apoE, apoH, LCAT, acute-phase proteins apoAIV, SAA, complement C3, several proteinase inhibitor proteins, including A1AT, inter alpha-trypsin inhibitor and α-2-macroglobulin were increased in SR-BI^−/−^ HDL, as compared to those in SR-BI^+/+^ HDL (Additional file 2: Table S2).

**Fig. 1:**
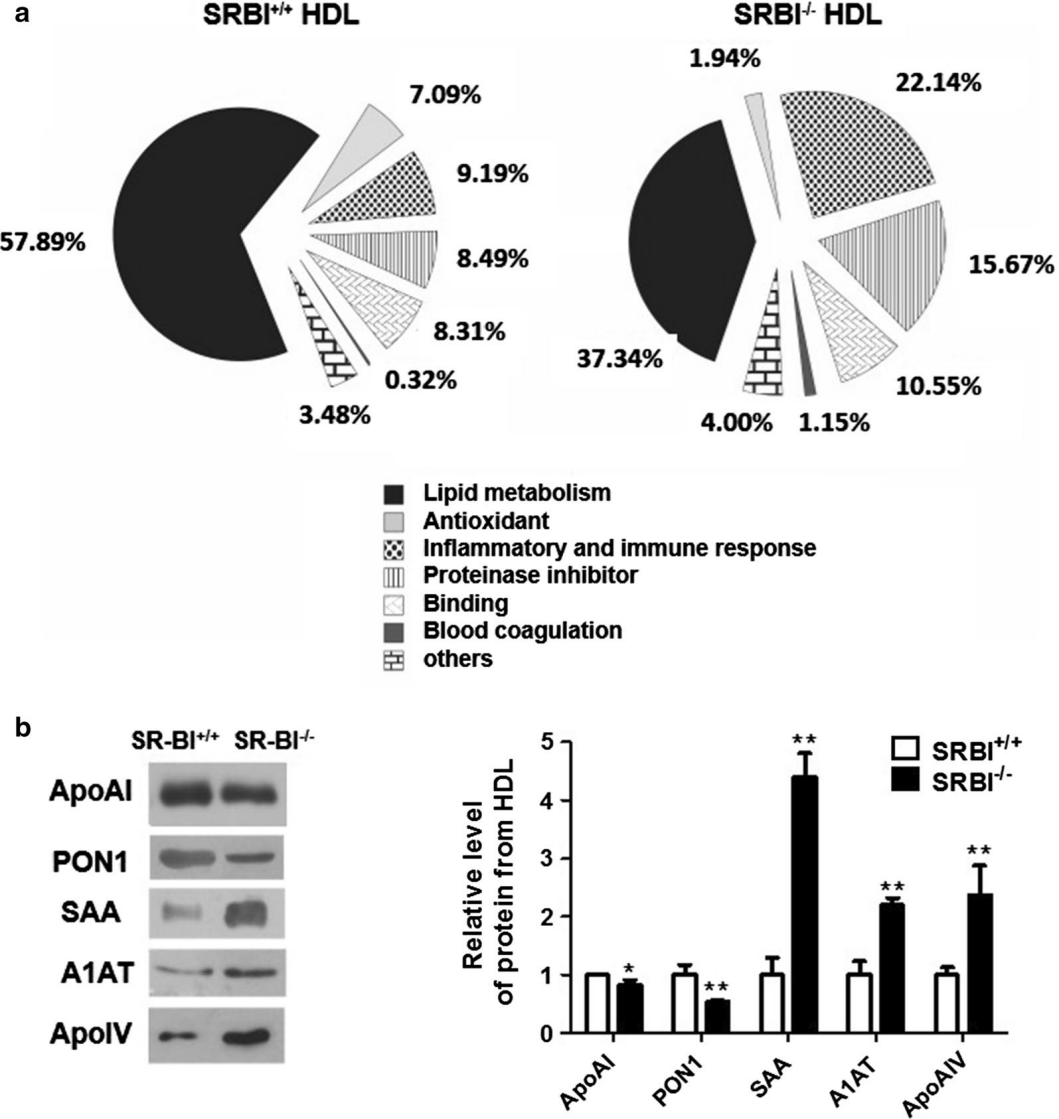
SR-BI deficiency results in altered HDL protein composition. **a** Comparison of functional protein classification between of SR-BI^+/+^ HDL and SR-BI^−/−^ HDL using spectrum counts analysis. **b** The levels of HDL proteins isolated from SR-BI^+/+^ mice and SR-BI^−/−^ mice were analyzed by Western blotting (n = 3. *P < 0.05, **P < 0.01 versus SR-BI^+/+^ mice)

Gene ontology classification was applied to estimate the contribution of various functional protein classes present in HDL. Compared to SR-BI^+/+^ HDL, HDL from SR-BI^−/−^ mice had a significant decrease in proteins involved in lipid metabolism (37.34% versus 57.98%) and antioxidant (1.94% versus 7.09%), while had a significant increase in inflammatory and immune response (22.14% versus 9.19%) and proteinase inhibition (15.67% versus 8.49%) (Fig. 1a).

To further validate the proteome results, Western blotting analysis of representative HDL proteins were performed and demonstrated that apoAI and PON1 levels were decreased, whereas apoAIV, A1AT and SAA levels were increased in SR-BI^−/−^ HDL (Fig. 1b).

### Differential profile of plasma HDL proteins

**Fig. 2:**
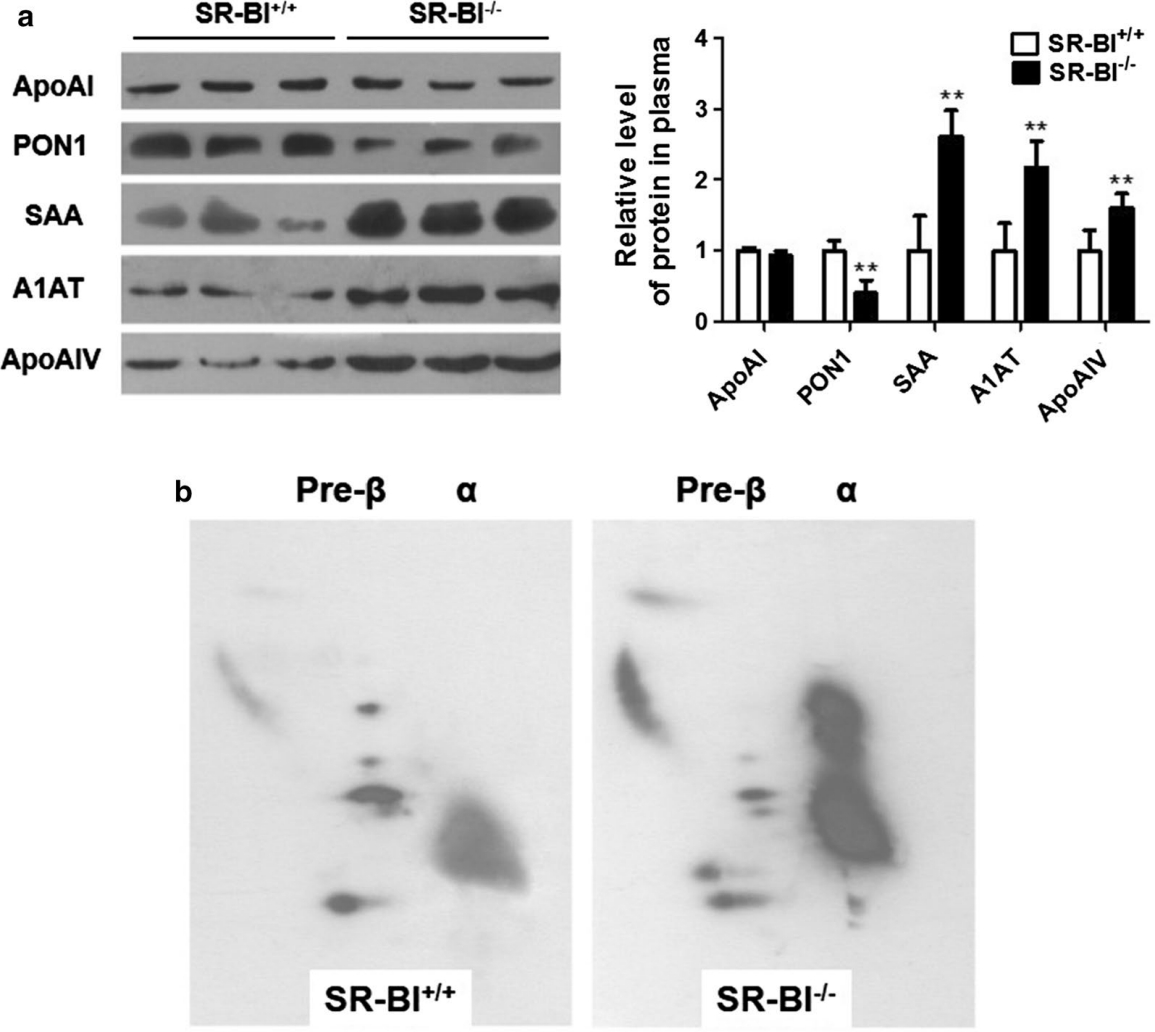
The differential profile of plasma HDL proteins composition in SR-BI^+/+^ mice and SR-BI^−/−^ mice. **a** Plasma HDL proteins levels were analyzed by Western blotting. n = 6, **P < 0.01 versus SR-BI^+/+^ mice. **b** Different distribution of apoAI-containing HDL subpopulations separated by 2D gel electrophoresis. The mobility of α and pre-β migrating particles (first dimension) were indicated

We further measured the levels of those proteins in mouse plasma, the data showed that the changes of PON1, SAA, A1AT and apoAIV levels in plasma were consistent with those in HDL, but levels of apoAI in plasma were only slightly reduced in SR-BI^−/−^ mice compared to SR-BI^+/+^ mice (*P *> 0.05) (Fig. 2a).

Using 2D gel electrophoresis, we investigated the effects of SR-BI deficiency on apoAI-containing HDL subpopulations in the plasma (Fig. 2b). The profile showed that the majority of apoAI in plasma of SR-BI^+/+^ mice was in α-mobility and relatively small amount in pre-β migrating HDL. Compared to SR-BI^+/+^ mice, SR-BI^−/−^ mice had an accumulation of large HDL migrating between pre-β and α, suggesting that the compositions and structure of apoAI-containing HDL in SR-BI^−/−^ mice was dramatically altered.

### Functional alterations of SR-BI^−/−^ HDL

#### HDL-mediating cholesterol flux in macrophage

**Fig. 3:**
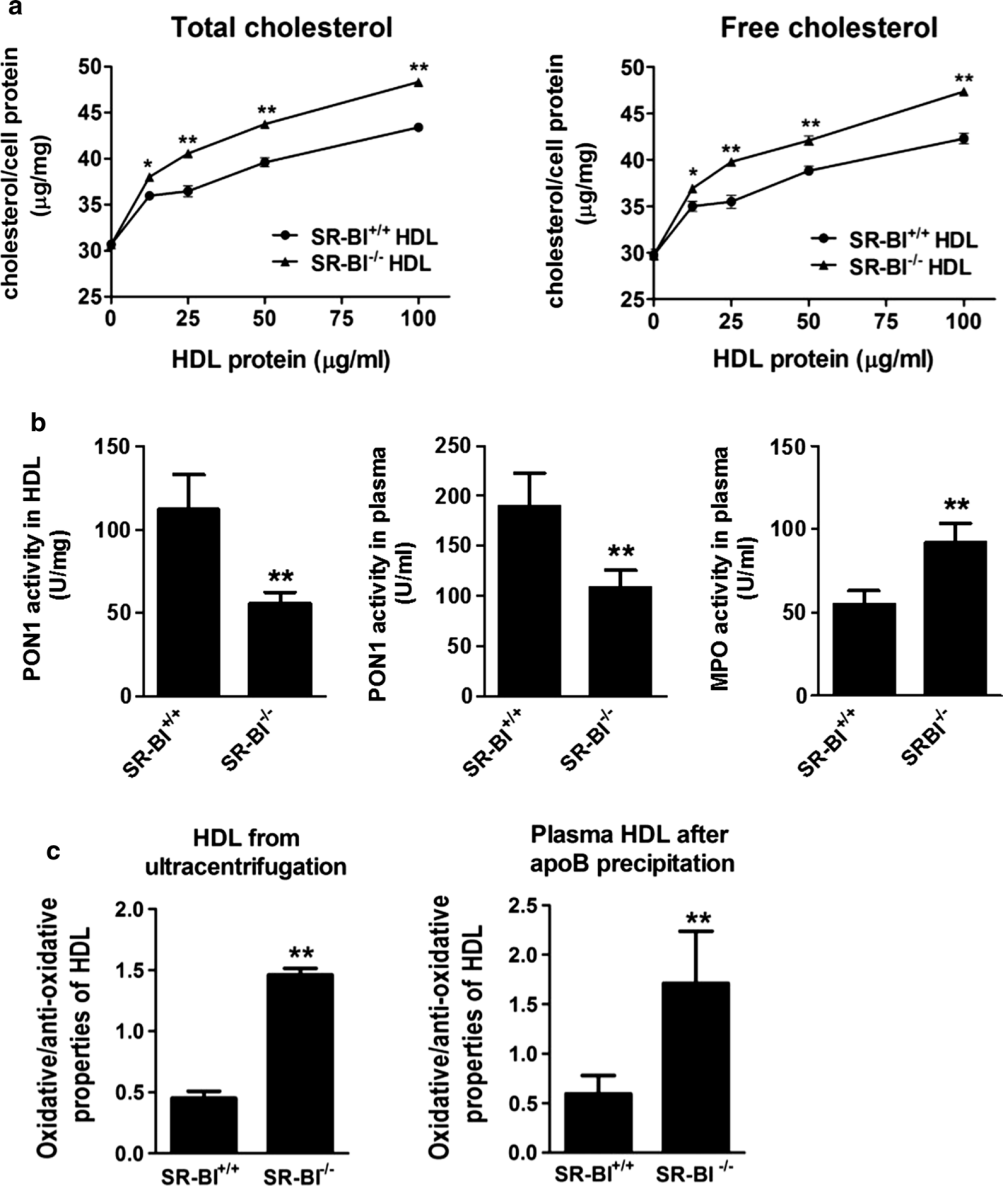
Comparison of HDL functions promotes more cholesterol accumulation in macrophage and reduced the anti-oxidative activity. **a** The levels of TC and FC contents in WT mouse macrophages incubated with SR-BI^+/+^ HDL or SR-BI^−/−^ HDL were examined. n = 3, **b** comparison of PON1 activity in HDL (n = 3) and in plasma (n = 20), and plasma MPO activity (n = 20). **c** Oxidative/anti-oxidative properties of HDL isolated by ultracentrifugation (left, n = 3) and plasma HDL (right, n = 8) were analyzed, *P < 0.05, **P < 0.01 versus SR-BI^+/+^ HDL. Values < 1.0 indicated anti-oxidative HDL, values > 1.0 indicated oxidative HDL

Considering that modification of apoAI and changes of HDL composition may impair the capacity of HDL in maintaining macrophage cholesterol homeostasis, we measured the cellular cholesterol content of WT macrophages after incubated with isolated HDL at various protein concentrations (12.5, 25 or 50 mg/mL) for 48 h. Compared to the cells treated with SR-BI^+/+^ HDL, the cells treated with SR-BI^**−/−**^ HDL had significantly more TC and FC remained in the cells in a concentration dependent manner (Fig. 3a).

#### Anti-oxidative activity of HDL

PON1, as a specific HDL-binding protein, contributes to the anti-oxidative activity of HDL. In agreement with proteomic data, PON1 activity was significantly reduced in both HDL and plasma of SR-BI^**−/−**^ mice (Fig. 3b). The PON1 activity in SR-BI^**−/−**^ HDL was decreased by 50% as compared with that in SR-BI^+/+^ HDL (55.7 ± 6.4 U/mg versus 111.9 ± 21.1 U/mg, *P *< 0.01), and plasma PON1 activity of SR-BI^**−/−**^ mice was significantly lower than that of WT mice (108.2 ± 17.5 U/mL versus 189.3 ± 33.8 U/mL, *P *< 0.01). Furthermore, we found that the plasma activity of MPO, an oxidative enzyme related to inflammation, was significantly higher in SR-BI^**−/−**^ mice.

In addition, the result of the oxidative/anti-oxidative properties of HDL detected by the cell-free assay showed that isolated HDL by ultracentrifugation or plasma HDL after apoB-lipoprotein precipitation from SR-BI^−/−^ mice had oxidative status with values > 1.0, compared with the anti-oxidative HDL from SR-BI^+/+^ controls, whose values were < 1.0 (Fig. 3c).

#### Anti-inflammatory capacity of HDL

**Fig. 4:**
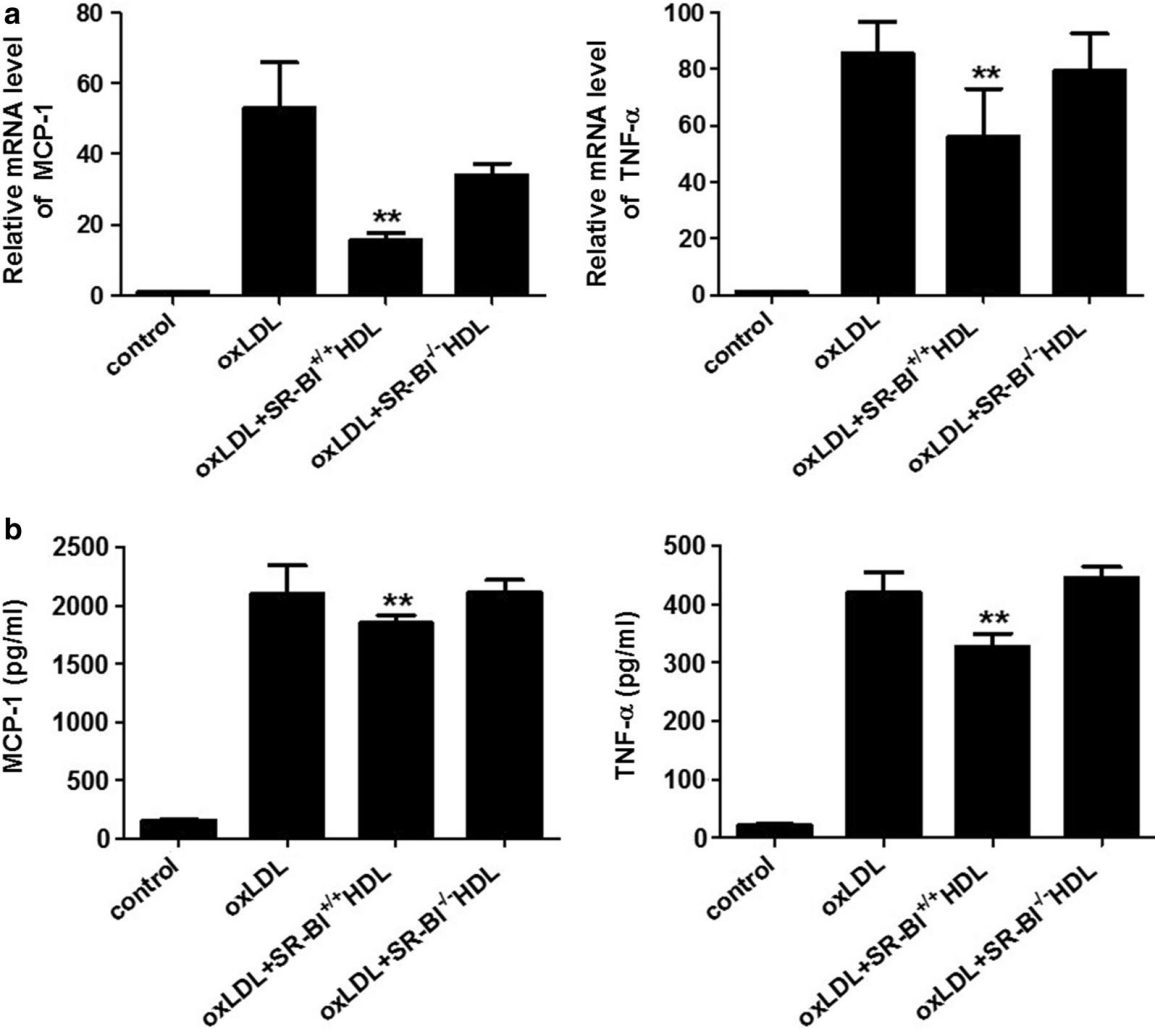
The effect of HDL isolated from mice on macrophage inflammatory response induced by oxidized LDL. The mRNA expression of MCP-1 and TNF-α in macrophages were measured by qPCR (**a**) and the cytokine concentrations in the medium were measured by ELISA (**b**), n = 3, **P < 0.01 versus ox-LDL group

Macrophages respond to ox-LDL stimulation by expressing and secreting inflammatory mediators. We tested the effects of SR-BI^+/+^ HDL and SR-BI^−/−^ HDL on ox-LDL-induced cytokine production in macrophages. As shown in Fig. 4a, b, ox-LDL resulted in a strong induction of both MCP-1 and TNF-α expression and secretion in macrophages, and SR-BI^+/+^ HDL significantly diminished the induction at both mRNA and protein levels, while SR-BI^−/−^ HDL showed no effects. This data suggests that SR-BI^−/−^ HDL lost its anti-inflammatory property.

### Remodeling structure and function of SR-BI^−/−^ HDL by probucol treatment

**Fig. 5:**
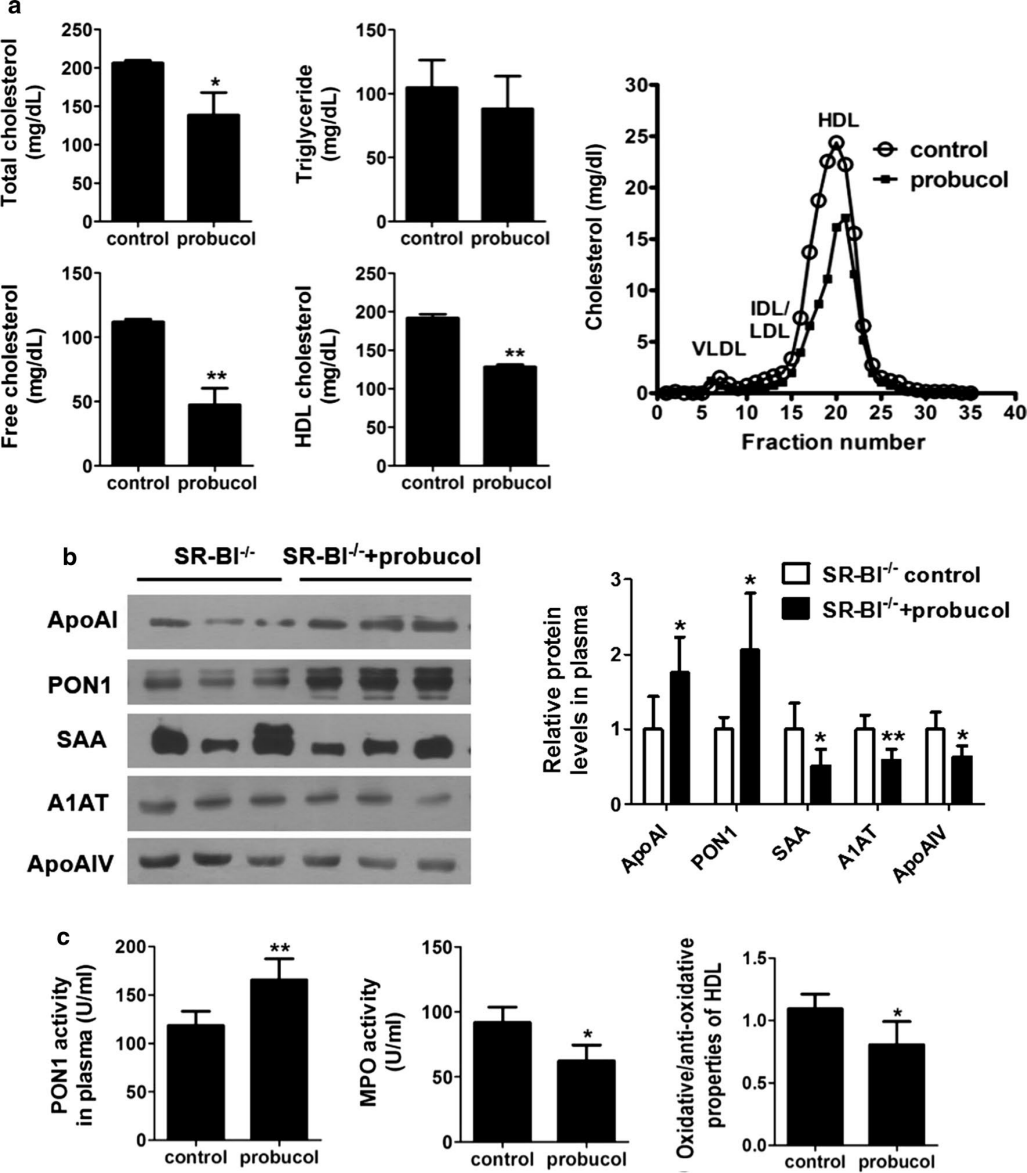
The effects of Probucol treatment on HDL lipid, proteins composition and functions in SR-BI^−/−^ mice. **a** The effects of probucol on plasma lipid levels and cholesterol profile separated by FPLC in SR-BI^−/−^ mice. n = 4. **b** Effects of probucol on HDL-associated proteins in SR-BI^−/−^ mouse plasma. **c** The effects of probucol on the function of HDL in SR-BI^−/−^ mice. n = 4. *P < 0.05, **P < 0.01 versus SR-BI^−/−^ mice group

After SR-BI^−/−^ mice were treated with probucol, plasma TC and FC levels were significantly decreased, mainly in the HDL-C fractions, compared with the control group (Fig. 5a). However, the HDL-binding proteins apoAI and PON1 levels in plasma were significantly increased, and acute-phase proteins apoAIV, SAA and A1AT levels were reduced in SR-BI^−/−^ mice treated with probucol (Fig. 5b); and the anti-oxidative function of HDL in SR-BI^−/−^ mice could be improved, evidenced by increases in plasma PON1 activity and decreases in HDL oxidative property and plasma MPO activity (Fig. 5c).

## Discussion

In current study, we demonstrate that the defective HDL functions in SR-BI^−/−^ mice are associated with the altered protein composition by shotgun proteomic profiling and biochemical analyses, despite the higher HDL-C level in SR-BI^−/−^ mice. We observed that a significantly lower abundance of lipid metabolism and antioxidant related proteins, such as apolipoproteins (apoAI, apoAII and apoCI) and HDL-associated PON1 in SR-BI^−/−^ mice, whereas levels of proteins involved in inflammatory and immune response, proteinase inhibition and blood coagulation, including SAA, apoAIV, complement C3 and A1AT, were increased. These protein composition alterations result in impairment of HDL function, including impaired function in maintaining cholesterol homeostasis in macrophages, and the anti-oxidative and anti-inflammatory capacity. Furthermore, the lipid-lowering and antioxidant drug probucol can improve the HDL functions in SR-BI^−/−^ mice by reversing the contents of some critical proteins, including increasing plasma apoAI and PON1 levels, and decreasing plasma SAA, apoAIV and A1AT levels.

There is accumulating evidence that alterations in HDL protein quality and composition result in HDL function impairment [24, 25]. In this study, we employed shotgun proteomics methods to identify the quantitative and qualitative alterations in HDL-associated proteins that may render SR-BI^−/−^ HDL dysfunctional. The proteomic profiling of HDL particles shown that some of essential protective apolipoproteins and enzymes were decreased, and the oxidative and inflammatory related proteins identified in SR-BI^−/−^ HDL appeared or were increased, compared to SR-BI^+/+^ HDL. We observed that relative levels of HDL-associated apolipoproteins involved in lipid metabolism, such as apoAI, apoAII, and apoCI, were significantly decreased in SR-BI^−/−^ HDL. As the key apolipoprotein on HDL, apoAI is a well-known anti-atherogenic molecule by mediating reverse cholesterol transport (RCT) and exerting anti-inflammatory effects [26–28]. ApoAII, as the second most abundant protein on HDL, also acts as a primary acceptor and efficiently removes cholesterol from macrophages in vivo [29]. ApoCI, as a component of HDL and VLDL, was considered to play a beneficial role in lipid metabolism, but recently study reported that apoCI could increase the LPS-induced inflammation in macrophages in vitro and in apoE^−/−^ mice [30]. In the proteomic profiling, we found that the relative abundance of apoCI in SR-BI^−/−^ HDL was decreased. Therefore, the role of apoCI in SR-BI^−/−^ mice remains to be further studied. An interesting finding is that the percentage of apoAI is decreased in HDL by spectral counting, while the plasma apoAI content is not reduced in SR-BI^−/−^ mice. Meanwhile, we also identified by 2D gel electrophoresis that the SR-BI^−/−^ mice had an accumulation of large HDL migrating between pre-β and α, with a higher level of apoAI. These suggested that apoAI in abnormal SR-BI^−/−^ HDL might have underwent dramatic modification, and became loosely associated with the larger HDL, thus, part of apoAI was likely to lost from isolation of HDL by precipitation and ultracentrifugation. Considering that SR-BI^−/−^ mice had comparable plasma apoAI concentration as WT mice (108 versus 100 mg/dL), as reported by a previous study [12], it is conceivable that the relative level of apoAI in SR-BI^−/−^ HDL was significantly reduced due to modification of apoAI and elevation of other proteins such as inflammatory proteins. Indeed, our results further confirmed that SR-BI^−/−^ HDL contributed to the accumulation of cholesterol in macrophages, indicating that SR-BI^−/−^ HDL not only stimulated FC influx towards the cells with gradient-regulated transport, but also impaired the ability of mediating cholesterol efflux because of modification of apoAI and other compositional alterations.

Besides involved in lipid metabolism, apoAI also exerts the anti-oxidative and anti-inflammatory functions of HDL. A recent study has demonstrated that impaired antioxidant HDL function is independently associated with the development of premature acute myocardial infarction [31]. In addition to apoAI, several important enzymes such as PON1, PON3 and PAFAH in HDL play anti-oxidative role. As an essential HDL-associated enzyme, PON1 contributes substantially to the anti-inflammatory properties of HDL through preventing LDL oxidation by hydrolyzing oxidized lipids. Our previous study has reported that lower plasma PON1 activity is associated with increased atherosclerotic lesions in apoE^−/−^ mice and in coronary artery disease (CAD) patients [21]. This present study showed that the abundance and activities of PON1 in HDL and plasma of SR-BI^−/−^ mice were markedly reduced, indicating that anti-oxidative activity of SR-BI^−/−^ HDL was hampered. Therefore, oxidative stress was increased in SR-BI^−/−^ mice, which is evidenced by increased plasma MPO activity and HDL-related oxidative property, consistent with a previous study [14].

We also found some other proteins which may contribute to the pro-inflammatory properties of SR-BI^−/−^ HDL, such as acute-phase protein SAA, apoAIV, several cell adhesion molecules, complement C3 and immune response proteins. SAA is demonstrated as a clinically useful marker for inflammation and is strongly associated with increased risk of cardiovascular events [32]. A previous report showed that SAA could result in altered metabolic properties of HDL by displacing apoAI, rendering HDL proatherogenic [33]. Human apoAIV has been reported with multiple functions related to lipid metabolism through activation of LCAT and modulation of lipoprotein lipase, and anti-oxidative activity [34]; however, mouse apoAIV, as a positive acute phase protein, was increased during inflammation [35]. Our study identified that SR-BI^−/−^ mice had higher HDL-associated apoAIV levels, supporting that SR-BI^−/−^ HDL in the inflammatory state. The previous study showed that SR-BI^−/−^ mice have a significant lower LCAT activity [36], Our results suggested that decreased activity of LCAT maybe related with apoAI modification and higher apoAIV levels in SR-BI^−/−^ mice, although the relative LCAT level of SR-BI^−/−^ HDL was increased in proteomic profile. In addition, proteins involved in cell adhesion like intercellular adhesion molecule 1 (ICAM1), integrin β1, and vitronectin were found in SR-BI^−/−^ HDL, but not in WT HDL. Several proteins with serine proteinase inhibitor domains were detected with increased levels in SR-BI^−/−^ HDL, such as A1AT, antithrombin-III, inter-α-trypsin inhibitor, angiotensinogen, α-2-macroglobulin and murinoglobulin-1, compared to SR-BI^+/+^ HDL. A recent study of HDL proteomics indicated that protease inhibition ability modulates the anti-inflammatory properties of HDL [37]. Our proteomic data supported that accumulation of the damaged proteins in SR-BI^−/−^ HDL could initiate inflammation, coagulation, and complement activation responses. Furthermore, we observed that HDL isolated from SR-BI^−/−^ mice exhibited less capability of suppressing inflammatory cytokine (MCP-1, TNF-α) expression induced by ox-LDL in macrophages. Taken together, the present study demonstrated that SR-BI deficiency results in a significant alteration in the HDL proteome, which contributes to impair the critical HDL functions, may explain increased risk of cardiovascular disease despite higher HDL-C level in SR-BI^−/−^ mice.

Certainly, mouse does not express CETP, which transfers cholesteryl ester from HDL to VLDL/LDL for further uptake by the liver, it means that HDL metabolism and its related proteins of mice are not exactly the same as those of humans. For example, the changes of apoAIV and A1AT in defective HDL in SR-BI^−/−^ mice were different from those in patients with HDL-associated metabolic diseases [35, 38]. However, the previous studies showed that dysfunctional HDL with increased plasma HDL-C levels in SR-BI^−/−^ mice mirrored that in those patients who harbor SR-BI mutations [9–11]. Accordingly, our observation made in SR-BI^−/−^ mice studies also supported that the identification of significant proteins in dysfunctional HDL using SR-BI^−/−^ mice as a model, might provide a powerful clue for exploring the biomarkers in clinical diagnosis and therapeutics of dysfunctional HDL-related metabolic diseases. SR-BI^−/−^ mice could be considered as a good preclinical animal model for studying the structure–function relationship of HDL.

Probucol is lipid-lowering and antioxidant drug that has been used in clinic for the treatment of CAD. It could promote RCT, normalize the FC level of HDL [39, 40], and reverse female infertility in SR-BI^−/−^ mice [16]. Therefore, we explored the effects of probucol on several critical HDL protein components and HDL dysfunction in SR-BI^−/−^ mice. The current study indicated that accompanied with decreased plasma TC, FC and HDL-C levels, the alterations of some representative proteins of SR-BI^−/−^ HDL could be reversible, and anti-oxidative function was also improved by probucol treatment. These are in agreement with the other studies, that small, protein-enriched, cholesterol-depleted HDL particles are more effective in mediating cholesterol efflux, and with more potent antioxidant, anti-inflammatory, and anti-apoptotic capacity [41, 42]. The derived protein biomarkers in dysfunctional HDL may eventually help to exploit diagnostics targets and therapeutic strategies for HDL-related metabolic diseases.

## Conclusion

Our proteomic and functional assessments of HDL provide evidence that defective HDL in SR-BI^−/−^ mice have significant decreased levels of apoAI and PON1 proteins and increased levels of pro-inflammatory proteins, such as SAA, apoAIV, and A1AT. The alterations in protein markers of SR-BI^−/−^ HDL result in the dysfunction of HDL including impaired capability in maintaining macrophage cholesterol homeostasis, and the reduced anti-oxidative and anti-inflammatory capabilities. These HDL dysfunctions in SR-BI^−/−^ mice could be improved by probucol treatment. This observations suggest that quantification of apoAI, PON1, SAA, apoAIV, and A1AT may serve as the valuable protein markers for diagnosis and therapeutics of dysfunctional HDL-related metabolic diseases.

## Additional files

**Additional file 1: Table S1.** Composition of HDL isolated from SR-BI^+/+^ and SR-BI^−/−^ mice.

**Additional file 2: Table S2.** Identification of HDL-associated proteins.

## Abbreviations

A1AT: α-1-antitrypsin
CAD: coronary artery disease
CE: cholesterol ester
CETP: cholesteryl ester transfer protein
FC: free cholesterol
FPLC: fast protein liquid chromatography
HDL-C: HDL-cholesterol
LC: liquid chromatography
MCP-1: monocyte chemotactic protein 1
MPO: myeloperoxidase
ox-LDL: oxidized LDL
PON1: paraoxonase-1
PEG: polyethylene glycol
RCT: reverse cholesterol transport
SAA: serum amyloid A
SR-BI: scavenger receptor class B type I
TC: total cholesterol
TNF-α: tumor necrosis factor α
2DE: 2D nondenaturing agarose-polyacrylamide gel electrophoresis
