# Supplementary material for: Protein markers of dysfunctional HDL in scavenger receptor class B type I deficient mice
Source: J Transl Med. 2018 Jun 7;16:155. doi: 10.1186/s12967-018-1502-y (PMC5992774; doi:10.1186/s12967-018-1502-y)
Supplement: Supplementary file 1 — Additional file 1: Table S1. Composition of HDL isolated from SR-BI+/+ and SR-BI−/− mice. [file 12967_2018_1502_MOESM1_ESM.docx]

**Table S1. Composition of HDL isolated from SRBI^+/+^ and SRBI^-/-^ mice.**

Values were means±SD of five independent experiments . ** *P*<0.01 as compared with SR-BI^+/+^ HDL
