# Supplementary material for: Protein markers of dysfunctional HDL in scavenger receptor class B type I deficient mice
Source: J Transl Med. 2018 Jun 7;16:155. doi: 10.1186/s12967-018-1502-y (PMC5992774; doi:10.1186/s12967-018-1502-y)
Supplement: Supplementary file 2 — Additional file 2: Table S2. Identification of HDL-associated proteins. [file 12967_2018_1502_MOESM2_ESM.docx]

**Table S2. Identification of HDL-associated proteins**

HDLs (1.09＜d＜1.21 g/ml) were isolated from fresh pooled plasma samples of SR-BI^+/+^ or SR-BI-/- mice by sequential ultracentrifugation. The HDL proteome was analyzed by LC-MS/MS, and data were analyzed by searching the mouse NCBI database with SEQUEST Algorithm version 4.0.3 (Sage-N Research, San Jose, CA). Values represent the percentage of peptides of total peptide count per analyzed subject. All data presented were the mean±SD value of three independent experiments.
